# The Major Facilitator Superfamily and Antimicrobial Resistance Efflux Pumps of the ESKAPEE Pathogen *Staphylococcus aureus*

**DOI:** 10.3390/antibiotics12020343

**Published:** 2023-02-07

**Authors:** Jerusha Stephen, Fathima Salam, Manjusha Lekshmi, Sanath H. Kumar, Manuel F. Varela

**Affiliations:** 1 ICAR-Central Institute of Fisheries Education (CIFE), Mumbai 400061, India; 2 Department of Biology, Eastern New Mexico University, Portales, NM 88130, USA

**Keywords:** multidrug resistance, *Staphylococcus aureus*, major facilitator superfamily, bacterial pathogens, multidrug efflux, transporter proteins, ESKAPEE

## Abstract

The ESKAPEE bacterial pathogen *Staphylococcus aureus* has posed a serious public health concern for centuries. Throughout its evolutionary course, *S. aureus* has developed strains with resistance to antimicrobial agents. The bacterial pathogen has acquired multidrug resistance, causing, in many cases, untreatable infectious diseases and raising serious public safety and healthcare concerns. Amongst the various mechanisms for antimicrobial resistance, integral membrane proteins that serve as secondary active transporters from the major facilitator superfamily constitute a chief system of multidrug resistance. These MFS transporters actively export structurally different antimicrobial agents from the cells of *S. aureus*. This review article discusses the *S. aureus*-specific MFS multidrug efflux pump systems from a molecular mechanistic perspective, paying particular attention to structure–function relationships, modulation of antimicrobial resistance mediated by MFS drug efflux pumps, and direction for future investigation.

## 1. *Staphylococcus aureus*

*Staphylococcus aureus* is a Gram-positive, commensal bacterium commonly inhabiting the human skin and mucosal tracts. This bacterium causes serious healthcare-associated (nosocomial) infections and is a significant cause of mortality and morbidity in hospital facilities [1]. Moreover, *S. aureus* is also frequently linked to food poisoning outbreaks associated with milk, eggs, and meat. It is considered the third most significant cause of food-borne infections worldwide, especially owing to its ability to produce heat-stable exotoxins [2].

The infections caused by *S. aureus* include bacteremia, endocarditis, skin and soft tissue infections, sepsis, central nervous system infections, urinary tract infections, pneumonia, toxic shock syndrome, osteomyelitis, and others. In nasal epithelial cells, *S. aureus* can remain persistently with the help of many proteins and other cell surface constituents. These bacteria are known to colonize the anterior nasal passages of 20% to 80% of the overall human population [3]. The high prevalence of antibiotic-resistant *S. aureus* in clinical and environmental settings poses an additional threat to human health. The exceptional ability of *S. aureus* to offer resistance to many common antibiotic groups, such as aminoglycosides, penicillins, macrolides, tetracycline, and others, is of great concern, especially in treating serious infections in humans [4]. Methicillin-resistant *S. aureus* (MRSA) includes a class of *S. aureus* that emerged due to the rampant use of methicillin and semi-synthetic anti-staphylococcal penicillins to treat *S. aureus* infections in the 1960s [5]. The *mecA* gene encodes methicillin resistance, and MRSA is of significant concern in environmental and community settings because of the high mortality associated with MRSA infections. 

## 2. *Staphylococcus aureus* Is an ESKAPEE Pathogen

The ongoing use of antibiotics and exposure to nosocomial infections have accelerated the emergence of multidrug-resistant bacteria, resulting in a significant burden on healthcare systems [6]. More than two million infections with antimicrobial resistance (AMR) and a death toll of 29,000 in the United States alone occur each year at an attributable healthcare cost of more than USD 4.7 billion [7]. Bacteria are intrinsically resistant to certain antibiotics, and a small proportion of environmental bacteria might harbor clinically relevant antimicrobial resistance genes and act as reservoirs of such genes. Such bacteria represent a minuscule percentage of natural bacterial populations and may not pose a significant threat to humans. However, selection pressure induced by antibiotics can eliminate susceptible bacteria and select resistant populations, leading to their proliferation and dissemination. The development of antibiotic resistance is directly related to the use of antibiotics, or more precisely, the overuse of antibiotics [8,9,10,11]. The difficulties of immediately detecting bacterial infections and AMR genes in clinical settings lead to the extensive use of broad-spectrum antibiotics, which only exacerbates the development of resistance [12]. The application of antibiotics in the large-scale agriculture and food production sectors for disease treatment and prophylaxis has furthered the development and emergence of resistance and enhanced the optimal environments for their spread, as well as the selection of resistance [13,14,15]. AMR is not currently being monitored systematically, particularly in developing nations, where more than two-thirds of the world’s population lives. The current information indicates that each year, hospital- and community-acquired AMR infections are responsible for about 33,000 deaths and 874,000 years of life lost due to impairment, resulting in a direct or indirect loss of USD 1.5 billion [16,17]. 

The term ESKAPEE collectively represents pathogens capable of escaping the biocidal action of antibiotics, possessing an array of pathogenicity mechanisms, enabling survival in the host and the environment, and enhanced transmission. The group encompasses multidrug-resistant, both Gram-positive and negative bacterial pathogens such as species of the *Enterobacter* genus, *Staphylococcus aureus*, *Klebsiella pneumoniae*, *Acinetobacter baumannii*, *Pseudomonas aeruginosa*, *Enterococcus faecium,* and *Escherichia coli* [14]. ESKAPEE pathogens are listed in the WHO’s 2017 list, which divides pathogens into critical- and high-priority categories, which require urgent attention, and medium-priority pathogens. The critical-priority list includes carbapenem-resistant *P. aeruginosa* and *A*. *baumannii,* along with extended-spectrum β-lactamase (ESBL) and carbapenem-resistant *K. pneumoniae* and *Enterobacter* spp. In contrast, the high-priority list consists of vancomycin-resistant *Enterococcus faecium* (VRE) and methicillin- and vancomycin-resistant *S. aureus* (MRSA and VRSA) [18]. Antimicrobials are rendered ineffective against these pathogens by biological mechanisms such as drug inactivation, modification of drug binding sites, changes in cell permeability, and mutation. The Gram-negative members of ESKAPEE pathogens produce enzymes that irreversibly inactivate β-lactam antimicrobials. The extended-spectrum β-lactamases (ESBLs) and carbapenemase-like metallo-β-lactamases (MBLs) are a few examples. In addition to blocking antibiotics, ESKAPEE bacteria can also create biofilms that physically interfere with the host immune system. More than 40% of infections in intensive care units (ICU) are caused by them, particularly in individuals with an impaired immune system [19,20]. They can survive in the hospital environment for a relatively long time and can be easily transmitted from one individual to another. 

ESKAPEE pathogens are commonly isolated from clinical settings where they are linked to many potentially fatal illnesses, including bacteremia, pneumonia, meningitis, urinary tract infections, and wound infections, particularly in intensive care units [21]. Hospital waste and sewage spills that are not properly disposed of can contaminate the environment and lead to the presence of ESKAPEE pathogens in irrigation water, groundwater, and surface water systems [22], landfills [23], and industrial and municipal wastewater systems [24].

## 3. The Major Facilitator Superfamily (MFS)

### 3.1. Discovery of MFS Transporters

The discovery of the major facilitator superfamily (MFS) began with the pioneering studies of Prof. Peter J. F. Henderson and colleagues, who first observed that sugar transporters from bacteria to humans were homologous, indicating a common ancestral origin [25,26]. Members of the MFS include passive transporters, such as uniporters, and secondary active transport systems, such as symporters and antiporters [27]. Passive transporters allow the movement of solutes across the membrane from regions of higher concentrations to regions of lower concentrations till equilibrium is established. On the other hand, active transporters can transport solutes against a concentration gradient using either the energy derived from the hydrolysis of ATP (primary active transport) or the energy derived from the difference in an ionic gradient across the membrane (secondary active transport). Secondary active transport is further classified into symport, in which the substrate and ion are moved across the membrane in the same direction, as in the case of sugar transport, or the opposite direction, as in the case of drug efflux [10]. Interestingly, individual proteins within the MFS group differ widely in their substrates, transporting structurally different sugars, amino acids, intermediate metabolites, ions, and antimicrobial agents [27]. 

As new proteins of the MFS were discovered and the genetic elements encoding them were sequenced, families and sub-families were formed, eventually encompassing thousands of superfamily members [27]. These MFS transporters and members of other transporter superfamilies are housed in a continually updated transporter classification database, the TCD, https://tcdb.org/ (accessed on 18 January 2023). According to the TCD, the MFS has the largest number of families and individual transporter members [28].

### 3.2. Early Studies on the MFS

Early investigators noted similarities in the amino acid sequences and predicted secondary structures of MFS proteins, suggesting a commonality in the mechanism of solute transport across the membrane. These and other studies on the known antimicrobial efflux pumps of the MFS showed that they originated from a common ancestor composed of six transmembrane domains [29]. A later duplication event involving the primordial protein provided additional structures that diverged into new functional features, such as substrate selection or binding [29].

Sequence comparisons revealed that transporters of the MFS shared a series of highly conserved amino acid sequence motifs [30,31,32]. One of these sequence motifs, called motif A, found in the loop between the second and third transmembrane domains of almost all members of the MFS, was shown to be an essential element in various transporters, including antimicrobial agent transporters [33,34]. Another highly conserved sequence, found in the fifth helix of antiporters, called motif C or the antiporter motif, of drug and multidrug efflux pumps of the MFS, was demonstrated through mutagenesis to be functionally important [31]. The structure formed by the residues of motif C forms the mechanical basis of a hinge that undergoes conformational twisting during antimicrobial translocation across the membrane.

The structural nature of the MFS transporters relied primarily on sequence-based secondary structure predictions [35,36]. Two major structural features emerged from these reports: multidrug efflux pumps that harbored 12 or 14 transmembrane domains [27,36,37,38,39,40]. The first antimicrobial efflux pump to be crystallized and its molecular structure elucidated was YajR, a transporter with functional influence involving a domain structure called YAM [41], formed by residues of motif A [42].

### 3.3. Energization

The first studies concerned with the driving forces for the MFS transporters revealed that both passive and active transport systems are responsible for substrate translocation across the membrane [27,37,40]. These MFS energization modes included uniporters, symporters, and antiporters [27]. The uniport proteins are driven primarily by the solute gradients. A historical example of a model uniporter of the MFS is GLUT1 [43]. On the other hand, the symporters and antiporters are ion-gradient driven, as predicted by Mitchell in several organisms, including *S. aureus* [44,45,46,47]. The lactose permease, LacY, from *Escherichia coli* is a well-known and highly studied proton-driven symporter [48,49]. Likewise, the antiport systems are driven by proton gradients, resulting in the active efflux of antimicrobial agents from cells such as *S. aureus* [50,51]. 

### 3.4. MFS Pump Structure

**Figure 1:**
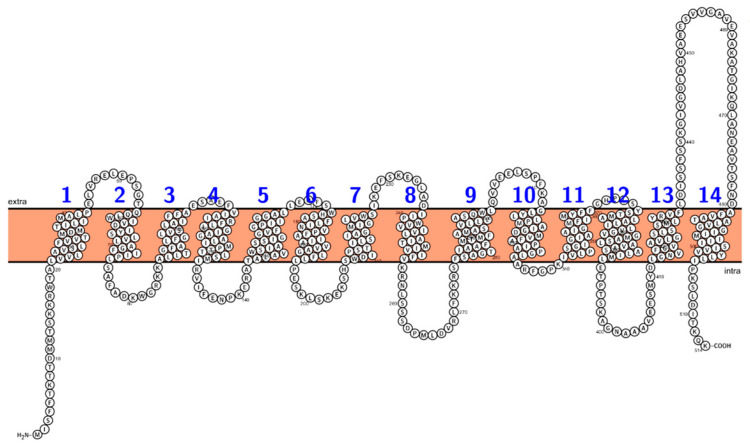
Topology of QacA. The transporters of the MFS have 12 to 14 transmembrane helices. Shown here is the two-dimensional structure of the multidrug efflux pump QacA, a well-studied MFS transporter from *S. aureus* [51].

One of the earliest crystal structures of an MFS multidrug efflux pump to be elucidated with high resolution, EmrD from *E. coli*, was reported in 2006 [52]. Additional MFS multidrug efflux pump structures include YajR, reported in 2013 [53], MdfA, discovered in 2015, and SotB, in 2021 [54], all from *E. coli*. In 2020, the crystal structure of the LmrP multidrug efflux pump from *Lactococcus lactis* was reported [55]. More recently, the NorC multidrug transporter from *S. aureus* was crystallized, and its structure was reported in 2020 [56]. In general, the multidrug transporters of the MFS share common features [57,58], Figure 1. Shared structural features of MFS antimicrobial transporters include 12 to 14 membrane-spanning segments that are α-helical, with a so-called canonical MFS-fold topology consisting of two bundles (N- and C-termini) composed of six or more transmembrane segments [57,58]. The MFS efflux pumps also harbor a large central flexible loop in the cytoplasmic side and a centrally localized cavity that is believed to alternately expose a binding site on either side of the membrane, participating in a so-called alternating-access mechanism [33,59,60]. 

In 2020, the structural determination of the MFS antimicrobial transporter NorC from the genome of *S. aureus* was published [56]. The NorC structure has 14 α-helices across the bacterial membrane. Moreover, the NorC crystal structure harbors an outward-facing conformation in an open state [56]. Interestingly, an antibody molecule was found within the extracellularly facing cavity, blocking substrate binding and transport [56]. The antibody–NorC complex and its effects on substrate accessibility can potentially be exploited to design new single-domain antibodies for other MFS multidrug efflux pumps of clinical relevance, such as antimicrobial pumps from ESKAPEE pathogens. 

In 2021, the crystal structure of SotB, a proton-driven drug efflux pump from *E. coli,* was determined with high resolution and bound to IPTG, forming a substrate-bound version in an inward-facing occluded state [55]. SotB harbors motifs A and C, implicating a translocation mechanism shared with other MFS transporters from *S. aureus* [55]. In 2022, using AlphaFold2 structure prediction software, it was reported that the various conformational structures that form between the N- and C-terminal bundles of MFS transporters could be studied for theoretical mutations with high fidelity [61]. The new structure prediction method may aid in studying solute transport dynamics, such as conformational changes of multidrug efflux pumps in bacteria [61]. 

### 3.5. Mechanisms of Antimicrobial Transport in MFS Efflux Pumps

**Figure 2:**
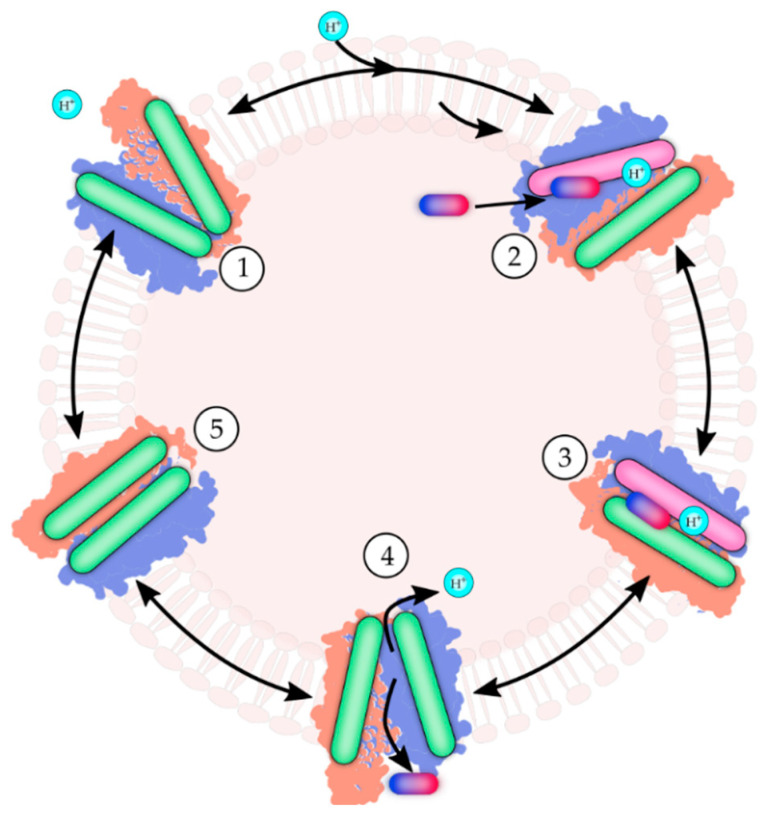
Ion-driven antiport mechanism of MFS multidrug efflux pumps. The oval cylinders represent the two N- and C-terminal bundles, surrounded by shaded purple and brown space-filling three-dimensional structures. Protons are denoted as encircled H^+^. The pink cylinder represents an enhanced affinity for the proton. The multicolored capsules depict antimicrobial agents. The mechanism described is based on studies summarized in references [62,63]. Steps 1–5 depict the following. 1: Outward open configuration of an empty MFS efflux pump. 2: The binding of H^+^ ion on the outside increases the drug binding affinity inside in an inward occluded state. 3: Intermediate occluded state. 4: Movement of the drug across the membrane to the outside and the H^+^ ion inside the bacterial cell. 5: Intermediate empty occluded state.

Based on early kinetics studies of secondary active drug transport across the membrane, a stepwise mechanism of drug efflux involving energization by ion gradients was proposed for proteins of the MFS [62,63]. The antimicrobial agent/ion antiport system, or efflux mechanism, is featured in Figure 2. The consensus antiport mechanism involves (1) an active energizing ion-motive force across the membrane and an empty MFS pump, followed by (2) an increase in the affinity of the pump for ion and an initial ion binding to the outward-facing efflux pump that is empty of the drug. The efflux pump’s initiating ion could be a proton or sodium [39,40]. The efflux pump experiences an inward-facing orientation that exposes the substrate-binding site and enhances the binding affinity for the antimicrobial agent. The antimicrobial agent binds the efflux pump, in which the drug-binding site is oriented inwardly, facing toward the cell’s cytoplasm. (3) A flexible conformation change transpires when the bound ion moves to an inward-facing and the bound substrate moves to an outward-facing orientation. The transporter is occluded or closed. (4) The antimicrobial substrate is released from the pump and diffuses to the outside of a Gram-positive cell. In Gram-negative bacteria, the substrate is released to the periplasmic space or an adaptor protein, followed by the exit through an outer membrane protein to reach the extracellular milieu of the bacterium. The ion is released from the pump and into the cell’s cytoplasm. The ion or substrate release order may differ depending on the particular efflux transporter studied. (5) The empty efflux pump reorients itself to switch the locations of the binding sites. In the case of MFS efflux systems, the ion binding site of the newly oriented pump now faces outward, and the antimicrobial agent substrate binding site faces inward. The reoriented antimicrobial efflux pump is ready to undergo another round of the transport cycle.

## 4. Structure and Function of MFS Multidrug Efflux Pumps in *S. aureus*

### 4.1. QacA

The QacA multidrug efflux pump system was discovered in 1989 [64,65]. Secondary structure analysis showed that the protein was highly hydrophobic and predicted to contain 14 transmembrane segments [66]. Evidence for the novel topology for QacA was demonstrated elegantly using fusions of truncated QacA fragments with reporter proteins [67]. A related MFS protein is the plasmid-based determinant encoding QacB, a multidrug efflux transporter from *S. aureus* [68]. The proton-driven QacA transporter enjoys a diverse array of structurally different antimicrobial substrates [69]. Interestingly, two conserved charged residues, Glu-26 and Asp-34, in helix one of QacA are thought to be involved in proton-coupled substrate transport [70]. The same study further demonstrated that QacA residues Glu-407 and Asp-411 participate in the proton-driven translocation of structurally different substrates through the pump [70]. As more clinical isolates of methicillin-resistant *S. aureus* are found with the *qacA* determinant, the QacA multidrug resistance system remains a relevant identification element and target for resistance modulation [71]. 

### 4.2. NorA

The NorA multidrug efflux pump was among the first antimicrobial transporters to be characterized extensively [72,73]. Early studies of the *norA* gene demonstrated resistance to norfloxacin, but the genetic element was later found to confer resistance to various structurally distinctive antimicrobial substrates in host cells. Of the many NorA-specific substrates, some include acridine orange, benzalkonium chloride, cetrimide ciprofloxacin, ethidium bromide, lomefloxacin, nalidixic acid, sparfloxacin, and tetraphenylphosphonium ion [74]. The NorA transporter is thought to harbor 12 transmembrane domains in the form of α-helices with a large centrally located loop between helices six and seven on the cytoplasmic side of the membrane [75]. Recent primary sequence analysis of homologs found that NorA and related transporters have novel conserved motifs called Motif-1 and Motif-2, which provide protein stability [76]. Molecular homology modeling of NorA structure offers a good system for screening putative efflux inhibitors.

### 4.3. TetA(K)

TetA typically refers to the antimicrobial efflux systems, while TetR denotes regulatory proteins. The genetic determinant for the TetA(K) (also denoted as Tet(K) and TetK) tetracycline resistance pump was discovered on an extrachromosomal plasmid, pT181, in isolates of *S. aureus* [77,78,79]. In 2014, a new series of tetracycline derivatives, e.g., omadacycline and aminomethylcycline, circumvented transport by TetA(K), suggesting their potential utility in restoring the clinical treatment of skin infections, especially those caused by MRSA [80]. Recently, the azoles, a class of antifungal agents, were shown to inhibit the transport of tetracycline through TetA(K) in *S. aureus* [81]. Further, when tetracycline was combined with butoconazole, clotrimazole, econazole, miconazole, or tioconazole, biofilm formation and antimicrobial activity were reduced [81]. 

### 4.4. Tet38

First reported in 2005, the resistance determinant encoding Tet38 was negatively regulated by MgrA, a multigenic regulator of gene expression [82]. Sequence analysis showed that the Tet38 protein is closely related to the TetA(K) tetracycline efflux pump, harbors 14 predicted transmembrane segments, and is a member of the MFS [82]. In addition to mediating resistance to tetracycline [83,84], tunicamycin [85], and Fosfomycin [86], Tet38 was demonstrated to transport fatty acids [87] and confer *S. aureus* internalization into host epithelial cells [88]. A more recent molecular analysis of Tet38 showed that various residues conferred differences in these functional characteristics [89]. For instance, mutations in residues in the extracellular loop between transmembrane helices 3 and 4 and a conserved residue of motif C in transmembrane helix 5 all result in a complete loss of resistance to tetracycline [89]. A deletion mutation in the loop between transmembrane helices 13 and 14 conferred a loss in cellular internalization. In contrast, a deletion in the loop between transmembrane helices 1 and 2 did not affect the host cell internalization of *S. aureus* [89].

### 4.5. MdeA

The MdeA multidrug efflux pump from *S. aureus* was discovered in 2004 and shown to be a member of the MFS [90]. The MdeA transporter has 14 predicted transmembrane segments and is known to confer resistance to structural derivatives of the quaternary ammonium compounds. Recently, the gene encoding MdeA was associated with serum resistance in clinical isolates from patients with poor prognosis following *S. aureus* bacteremia [91]. Speculatively, the MdeA efflux pump of *S. aureus* could play a role in expelling into the serum antimicrobial agents such as the quaternary ammonium compounds, novobiocin, mupirocin, and norfloxacin during bacteremia [91]. As mentioned above, MdeA is overexpressed in a clinical *S. aureus* isolate but downregulated by flavonolignans and chloramphenicol [92].

### 4.6. SdrM

In 2006, the SdrM efflux pump system was discovered in the laboratory of Tsuchiya [93]. The SdrM pump conferred resistance to acriflavine and norfloxacin and could readily transport ethidium bromide [93]. Hydropathy analysis of SdrM showed a hydrophobic protein with 14 transmembrane domains. Tsuchiya and colleagues demonstrated that SdrM is proton-driven, as exemplified by sensitivity to CCCP [93]. Interestingly, byproduct extracts harboring phenolic blueberry pomace from *Vaccinium corymbosum* or blackberry pomace from *Rubus fruticosus* showed downregulation of gene expression for the SdrM pump [94]. The SdrM multidrug efflux pump from *S. aureus* remains a poorly understood transport system and, hence, represents a promising area of investigation. 

### 4.7. LmrS

In 2010, our laboratory discovered the LmrS multidrug efflux pump system in an MRSA clinical isolate [95]. We demonstrated that the LmrS transporter actively effluxed ethidium bromide and conferred resistance to several structurally distinct antimicrobial agents, such as linezolid, lincomycin, florfenicol, gatifloxacin, trimethoprim, and tetraphenylphosphonium chloride [95]. LmrS is predicted to harbor 14 transmembrane domains [48]. As the transport activity of LmrS was sensitive to protonophores, we noted that the transporter is proton-driven [34]. The TetR21 repressor protein regulates the gene expression program for LmrS [96]. In addition to regulation of LmrS gene expression, it was recently shown that efflux pump activity for ciprofloxacin was inhibited by Plantaricin A (PlnA), an antimicrobial peptide isolated from *Lactobacillus plantarum* [97]. This study demonstrated a synergistic effect of transport activity when PlnA was combined with ciprofloxacin [97]. This antimicrobial efflux pump remains relevant as an indicator of virulence in clinical isolates and as a candidate for transferring its genetic determinant among diverse animal hosts and other microbial species. Recently, in a pathogenic clinical isolate of a coagulase-positive *S. aureus* from a patient with chronic respiratory failure, LmrS and other MFS multidrug efflux pumps, such as MdeA, NorA, NorB, and NorC, were found to be overexpressed [92]. Interestingly, the expression of the latter MFS multidrug efflux pumps, not LmrS, were sensitive to regulators such as chloramphenicol and the flavonolignans 2,3-dehydrosilybin B or 2,3-dehydrosilybin AB [92,98]. Thus, LmrS remains a serious *S. aureus* virulence factor that needs modulation to reduce conditions that foster virulence.

### 4.8. Other S. aureus MFS Multidrug Efflux Pumps

In 2005, a gene encoding the FexA efflux pump belonging to the MFS was found on a Tn*558* transposable element and transferred between various species of the *Staphylococcus* genus [99]. The gene encoding FexA was previously located on a plasmid from a bovine isolate *S. lentus* [99]. The substrate profile for FexA appears to be limited to the phenicol class of antimicrobial agents, such chloramphenicol and florfenicol, which also play gene-regulatory roles [99]. 

In 2012, a novel plasmid-encoded determinant Tet(63) from *S. aureus* was discovered [100]. Comparative sequence analysis revealed relatedness to TetA(K) with 14 predicted membrane-spanning segments [100]. The Tet(63) protein is a member of the MFS of transporters and confers resistance to tetracycline and doxycycline while reducing bacterial susceptibility to minocycline [100]. While tetracycline transport activity was not directly measured, the Tet(63) pump is sensitive to efflux pump inhibitors such as CCCP and reserpine, reducing MIC values. Interestingly, the Tet(63) determinant on the plasmid is located adjacent to a cluster of distinct antimicrobial agent resistance genes, implicating concomitant transfer of the *tet*(63) determinant to other species by selective pressure imposed from exposure to other antimicrobials [100]. 

In 2015, two *S. aureus* genomic loci encoding MFS transporters, SfaA and SbnD, were strongly implicated in the secretion of the iron-scavenging siderophores staphyloferrin A and B, respectively, into the extracellular milieu [101]. Hydropathy analyses predicted that SfaA has 12 transmembrane domains, while SbnD is predicted to have 10 such domains. Interestingly, the genetic determinants encoding SfaA and SbnD are located within regulated operons on the *S. aureus* genome, suggesting a means for selectively regulating the expression of these MFS efflux pump systems [101]

## 5. Role of Conserved Amino Acid Sequence Motifs

### 5.1. Motif A

The highly conserved amino acid sequence motif A was discovered in the late 1980s by Henderson and collaborators after conducting comparative gene sequence analyses [30,34]. Originally denoted as the consensus sequence “G (X)3 D R/K X G R R”, the residues of motif A are known to reside within the cytoplasmically located loop between transmembrane helices two and three of the great majority of solute transporters of the MFS [26,102]. Motif A has been the focus of intensive structure–function studies. Among the biological roles of the motif, key ones include a gating function [102], transporter structure stabilization using salt bridges, interface surfaces between N- and C-terminal domains [53], an electrochemical potential sensing system [103], and a conformational change regulator [104]. Recent reports provide evidence that residues of the signature sequence of motif A play a critical role in regulating the mechanism that controls transporter conformational switching, which occurs during substrate transport across the membrane [104]. 

### 5.2. Motif C

The highly conserved signature sequence of motif C, known as the antiporter motif, was discovered in 1990 by Skurray and colleagues [105]. The consensus sequence that was originally reported was “G (X)_8_ G (X)_3_ G P(X)_2_ G G”, which was located in the fifth transmembrane helix of antimicrobial efflux pumps of the MFS but absent in symporters and uniporters of the superfamily [31,105]. The first evidence for the functional importance of motif C involved a mutational analysis in which the most highly conserved residue of the signature sequence, Gly-147, in the TetA(C) efflux pump was converted into all other residues with a significant loss of tetracycline resistance [31,32,34]. The only acceptable replacements for Gly-147 were serine and alanine [31]. A molecular mechanics analysis showed a kinked structure forming in the fifth helix of the wild-type Tet(C) efflux pump [31]. The functional importance of additional residues of the motif was demonstrated in other antimicrobial efflux pumps such as TetA(K) [105] and TetA(L) [106,107], and multidrug efflux pumps QacA [108], VAChT [109], VMAT2 [110], and CaMdr1p [111], among others, reviewed elsewhere [112,113,114]. Additionally, crystal structures of multidrug efflux pumps display the kinked nature of the fifth helix that harbors motif C, such as YajR [53], VMAT2 [110], and MdfA [54]. These reports suggest that the structure determined by motif C’s residues forms a universal physiological basis in MFS pumps. The antiporter motif signature sequence plays a functional role as part of a molecular hinge that permits conformational changes to proceed during multidrug transport across the membrane [112]. 

## 6. Modulation of *S. aureus* MFS Multidrug Efflux Pumps

**Figure 3:**
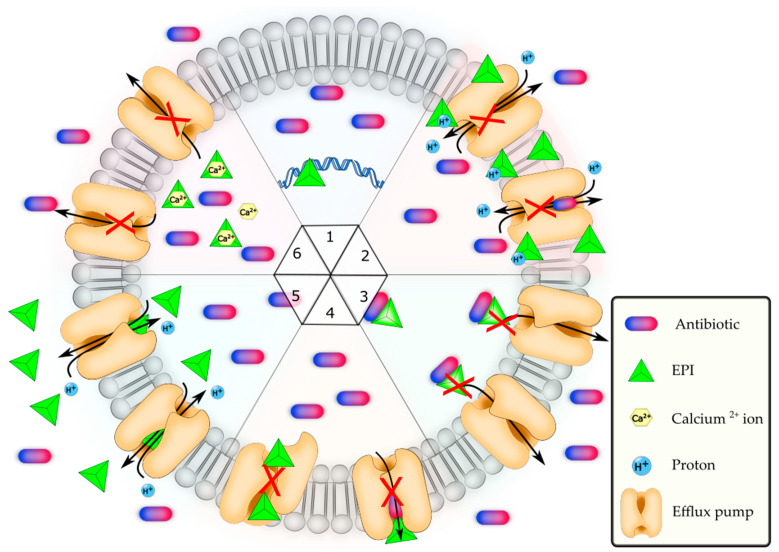
Modulation of MFS efflux pump by efflux pump inhibitors (EPIs) in *S. aureus*. 1: Downregulation of genes encoding an efflux pump. 2: Disruption of the membrane potential. 3: Interaction of EPIS with antimicrobials. 4: Disruption and impediment of efflux pump assemblies and membrane-bound proteins. 5: Competitive and non-competitive inhibition by EPIs. 6: Chelation of Ca^2+^ ions by EPIs in some specific efflux pumps, such as LmrS of *S. aureus* [117].

MFS efflux pump inhibitors (EPIs) derived from synthetic and natural sources have received much attention [115]. Another review [116] discusses a detailed list of substrates and EPIs for different MFS pumps of *S. aureus*. The mechanism of EPI inhibition may vary, but the most widely understood EPI mechanisms are shown in Figure 3.

### 6.1. Downregulation of Genes Encoding MFS Efflux Pumps 

The downregulation of efflux pump genes can negatively modulate the rate at which antimicrobial compounds are extruded from the bacterial cell. Recently, green nanoparticles have been shown to have the ability to downregulate efflux pumps. Samarium oxide nanoparticles prepared with curcumin reduced the expression of the *norA* and *norB* genes by 40% and 30%, respectively [118]. The nanoparticles also inhibited the ability of *S. aureus* to form biofilms by 53%. The antibiotic-induced gene expression of MATE, MFS, and ABC efflux pumps was downregulated by 2,3-dehydrosilybin B and AB compounds extracted and purified from silymarin (milk thistle *Silybum marianum*) [92]. The bioactive extracts of berry pomace reduced the *norA, norB, norC, mdeA, sdrM,* and *sepA* genes [94]. The dihydroquinazoline analogs inhibited the NorA efflux pump, resulting in a 16-modulation-fold reduction in the MIC of norfloxacin. 

In sub-inhibitory concentrations, dihydroquinazoline analogs downregulated the *norA* gene, and when combined with norfloxacin, a 3-log reduction in the number of intracellular *S. aureus* in human monocytes was observed [119]. The antifungal compound ketoconazole could reduce the expression of *norA, norB,* and *norC,* resulting in an eight-fold decrease in the MIC [120]. Recent studies revealed that the vinyl halogenated fatty acids and nanoparticles such as iron oxide (Fe_3_O_4_) magnetic nanoparticles functionalized by glutamic acid and conjugated with thiosemicarbazide [121], Fe_3_O_4_@Ag nanocomposite by *Spirulina platensis* [122], and ZnO nanoparticles conjugated to thiosemicarbazide under amine functionalization by glutamic acid [123] could downregulate the expression of the *norB* gene. The plant metabolite clerodane diterpene downregulated the expression of *norA, norB, norC, mepA,* and *mdeA* genes [124].

### 6.2. Disruption of the Membrane Potential 

Many MFS efflux pumps are secondary active solute transporters that utilize an ionic gradient (H^+^) to transport substrates across the membrane. Thus, the collapse of a proton motive force hinders the efflux of antibiotics. The carbonyl cyanide *m*-chlorophenylhydrazone (CCCP) is a well-studied uncoupler of the proton potential [125]. Carnosic acid, a diterpene found in herbs such as rosemary and sage, can inhibit the NorA efflux pump by causing the membrane potential to collapse, resulting in an eight-fold reduction in the MIC of erythromycin [126]. 

### 6.3. Interaction of EPIs with Antibiotics

This mechanism is based on the hypothesis that certain EPIs have an affinity towards the antimicrobial substrates of efflux pumps, and the binding of an EPI with the antibiotic results in a large inhibitor–drug complex that is unsuitable as a substrate for efflux pumps [127,128]. This potential mechanism has not been demonstrated in *S. aureus* MFS pumps.

### 6.4. Disruption and Impediment of Efflux Pump Assemblies and Membrane-Bound Proteins 

Tannic acid inhibited the NorA efflux pump by disrupting membrane-bound proteins [129]. The astringent complexion with microbial enzymes and substrates, the disruption of membrane potential, and the chelation of metal ions required for the efflux pump have been hypothesized to be the possible mechanisms of efflux inhibition by tannins [130].

### 6.5. Competitive and Non-Competitive Inhibition by EPIs 

EPIs may inhibit antimicrobial efflux in a competitive or non-competitive manner. The EPI has a higher affinity with the efflux pump during competitive inhibition, compromising the efflux of the antimicrobial compound. The chalcones extracted from *Arrabidaea brachypoda* reduced norfloxacin efflux via competitive inhibition, resulting in a four-fold reduction in the MIC. The docking study revealed that the molecule interacted with the NorA efflux pump’s Ser-218, Ile-135, Thr-335, and Asn-339 residues [131]. Thiophene, a curcuminoid, showed competitive inhibition against norfloxacin, and formed an H bond with the Arg-310 residue of NorA [132]. The furanochromones khellin and visnagin showed EPI activity by interacting with the Arg-310 residue of the NorA efflux pump, resulting in a two-fold reduction in the MIC of norfloxacin [132]. The in silico docking method revealed a possible competitive inhibition mechanism, as the furanochromones hinder the binding of EtBr [132]. Similarly, the aminoguanidine hydrazones, a series of biosynthetic agents, exhibited competitive inhibition of the NorA efflux pump [133,134]. 

### 6.6. Ca^2+^ Chelating EPIs

Calcium ions (Ca^2+^) play important roles in various signaling pathways, particularly in sensing environmental stressors. Nava et al. [117] discovered that the *S. aureus* intracellular Ca^2+^ concentration rapidly varied when exposed to various antibiotics, such as erythromycin, gentamycin, kanamycin, vancomycin, streptomycin, and ciprofloxacin [117]. Furthermore, different antibiotics tested elicited distinct Ca^2+^ transients, indicating that *S. aureus* can detect and distinguish between different antibiotic classes. The *lmrS* gene was then cloned and expressed in *E. coli* DH5 to investigate the modulatory role of Ca^2+^ on the LmrS efflux pump. The addition of Ca^2+^ chelators phenothiazine chlorpromazine (CPZ) verapamil and ethylene glycol-*bis*(β-aminoethylether)-N,N,N′,N′-tetraacetic acid (EGTA) significantly reduced efflux activity [135]. When Ca^2+^ was added, the reduced efflux was reversed. The functional role of divalent cations and their modulatory relationship with the MFS multidrug efflux pumps remain promising areas of investigation.

## 7. Inhibition of MFS Efflux Pumps of *S. aureus*

### 7.1. NorA, NorB and NorC Efflux Pumps

NorA is one of the most well-studied MFS pumps in *S. aureus*. Apart from its role in antimicrobial resistance to fluoroquinolone antibiotics, the efflux pump NorB plays a crucial role in bacterial fitness and survival in staphylococcal abscesses [136]. As reported in 2006 [137], the NorC X-ray structure has been recently elucidated [56]. The study revealed the interaction of tetraphenylphosphonium cation with NorC, though it cannot transport the molecule, as mentioned above. This finding can help in identifying potential NorC-specific EPIs [56]. 

Various plant-based compounds and essential oils have been shown to increase the susceptibility of the NorA-overexpressing strain of *S. aureus* to fluoroquinolone antibiotics [89,138,139,140,141,142,143]. Chalcones are unsaturated ketones that occur naturally in some yellow-pigmented flowers, the EPI potential of which has been well documented, particularly against the NorA efflux pump [129,144]. The NorA inhibition by chalcones and other EPIs seems to be strongly influenced by the degree of hydrophobicity, lipophilicity [144], and methylation [144], and methoxy group [145]. The crucial pharmacophores for NorA EPI are given as being a hydrogen bond acceptor, having a positive charge, presence of aromatic rings, and a hydrophobic region [146]. The EPI activity of terpenes such as eugenol and derivatives [146] showed that carvacrol and thymol modulation [147] could be due to competitive inhibition through hydrophobic lipophilic interactions and H bond formation. The α-bisabolol sesquiterpene from *Matricaria chamomilla* L reduced the norfloxacin MIC from 256 μg/mL to 32 μg/mL, although its mechanism of action is unclear. It is proposed that the EPI property could be due to competitive/non-competitive inhibition or the disruption of membrane potential [148]. 

The amino acid residues Arg-310 and Gln-51 in the binding pocket of the NorA efflux pump seem to be important amino acid residues for substrate recognition, and many EPIs interact with them through a hydrogen bond [149]. Imidazolines [150], imidazolidines [151], and aminoguanidine hydrazones EPIs [133] also interacted with the Arg-310 residue. The Arg-144 and Glu-126 residues that line the central pockets also seem to be the target for various EPIs [152]. The 2-(2-aminophenyl) indole (RP2) metabolite from soil microbe *Streptomyces roseochromogenes* inhibited the NorA efflux pump, resulting in a 64-, 16-, 4-, and a 4-fold reduction in the MIC of the tested antibiotics norfloxacin, ciprofloxacin, moxifloxacin, and chloramphenicol, respectively [153]. A docking study revealed an H bond formation with the Gln-51 residue, yet the mechanism of inhibition is unclear. It is hypothesized that the RP2 compound might act similarly to other EPIs, such as eugenol, totarol, thioxanthene, and phenothiazine [153]. A compound called phyllanthin extracted from *Phyllanthus amarus* resulted in a five-fold decrease in the norfloxacin MIC while creating an H bond with the residues Gln-51 and Asp-510 of the NorA efflux pump [154]. Coumarin derivatives docked with the NorA efflux pump interacted with the Lys-316, Asn-307, and Lys-273 residues, resulting in an eight-fold MIC reduction against norfloxacin [141]. Docking studies have shown that the amide group of synthetic riparin could form H bonds with the amide oxygen of Asn-340 and amide nitrogen of Gln-51, stabilizing the docking [144]. The inhibitory effect of capsaicin on NorA could be attributed to interaction with Pro-24, Phe-140, Ile-244, Gly-248, Phe-303, and Arg-98 residues, yet the exact mechanism of inhibition remains unclear. Interestingly, not only a reduction in fluoroquinolone MIC was noticed, but also a reduction in the emergence of ciprofloxacin-resistant mutants [155]. 

### 7.2. MdeA and LmrS

Inhibition of the LmrS pump using naturally occurring compounds extracted from the cumin spice *Cuminum cyminum* has been reported from our laboratory [95,156]. The transcriptional regulator TetR21 repressed the expression of Tet(38) and the LmrS efflux pumps [96]. The recent findings on the role of Ca^2+^ ions in the modulation of the LmrS efflux pump could lead to the identification of novel EPIs [117]. Except for the fluoroquinolones, overexpression of the MedA efflux pump confers resistance to quaternary ammonium compounds and some antibiotics [90]. Another EPI, piperine, reduced the MIC of mupirocin through direct efflux pump inhibition and reduced the mutation frequency [157].

### 7.3. Tet38 and TetA(K)

TetA(K) was one of the first MFS efflux pumps characterized in *S. aureus* [158]. In a study on MRSA strains, the tetracycline MIC was reduced 3 to 16 times with eugenol as an EPI [159]. Many other terpinenes are reported to be inhibitors of the Tet(K) efflux pump, such as the monoterpene α-pinene [160,161] and the combination of α-terpinene with essential oil from *Chenopodium ambrosioides* [162]. The flavonoid quercetin modulated the NorA and Tet(K) pumps, reducing the MIC of erythromycin, tetracycline, and norfloxacin. The interaction of quercetin with Ser-138 of NorA was observed, suggesting that hydroxyl groups of the flavonoid could stabilize the interactions [163]. Glycerol-3-phosphate exhibited a modulatory effect on the Tet38 pump concerning fosfomycin resistance [164].

## 8. Future Directions

The MFS antimicrobial efflux pump systems represent clinically significant virulence factors accounting for the compromised efficacy of chemotherapy for *S. aureus* infection [165]. These transporters thus represent suitable targets for modulation, either by direct efflux inhibition or gene expression regulation of the genetic determinants that encode the MFS multidrug efflux pumps [117,166]. Furthermore, the relationship between biofilm and bacterial residents with antimicrobial efflux pump systems represents an interesting field of investigation [167,168].

In particular, areas of research that need closer inspection include whether a universal mechanism of antimicrobial transport across the membrane is shared amongst MFS members of the ESKAPEE group and other pathogens of clinical importance. Much work is needed to understand whether such universality is a regulatable process and, thus, a suitable target for virulence modulation.

Another poorly understood area is how MFS transporters, such as uniporters and antiporters, convey a promiscuous efflux of structurally diverse antimicrobial agents while simultaneously preventing the leakage of ions, such as protons or sodium, and water, which would collapse the pumps’ energizing forces. Once investigators understand how these gating systems operate at the molecular physiological level, it would become possible to regulate these systems to drive more effective bacterial inhibition outcomes to restore clinical efficacy for older antimicrobials that have been compromised due to multidrug resistance, especially for ESKAPEE pathogens.

A related topic that needs attention is understanding how substrate specificity profiles are determined for a given multidrug efflux pump and how these substrate profiles are dictated. Once we understand more fully how the MFS drug exporters determine their substrate profiles for transport, then we can interrupt these systems to reduce pathogen resistance to multiple antimicrobials in a universal way, perhaps by the design of putative universal efflux pump inhibitors that target all the MFS of a particular pathogen, such as *S. aureus*.

Another poorly understood process regarding the function of MFS transporters is how they are energized to drive the transport. We do not yet have a clear handle on the MFS efflux pumps driven by substrate and ion gradients and how their transport function is tied to these energy-driving systems. Along these lines, it would be advantageous to gain a mechanistic foothold on how these energetic systems facilitate substrate translocation while undergoing the conformational changes that we know are occurring during transport. Indeed, it would be quite interesting to understand how ion selectivity operates in these ion antiporters of the MFS and whether the well-known mechanisms in classical ion channels can be applied to transporters of the MFS. On a related note, in several cases, the lactose permease LacY of *E coli*, a symporter, was converted into a passive transporter by single-point mutations [48,169].

The question of how much a single-substrate efflux pump can be altered by mutation to convert it into a multidrug efflux transporter remains unanswered. For the tetracycline transporters, TetA of the MFS [170,171], which are related phylogenetically to multidrug transporters, it is predicted that such a conversion is readily possible. Indeed, with sugar transporters of the MFS, substrate recognition could be altered by minor changes in sequence [48]. Thus, the alteration of antimicrobial specificity in MFS antimicrobial exporters to accommodate new or desired substrates would be important for target modulation and design. Such innovative studies as these and those designed to discover novel targets for modulation could be used to develop new anti-infective agents [167].

## Data Availability

Not applicable.
